# Globalizing digital immunization systems for the sustainable development goals: a perspective

**DOI:** 10.3389/fdgth.2026.1687302

**Published:** 2026-03-03

**Authors:** Sunny Ibeneme, Sean Blaschke, Khin Devi Aung, Benson Droti, Ridwan Gustiana, Hillary Kipruto, Basil Rodriques

**Affiliations:** 1 Harvard T.H Chan School of Public Health, Boston, MA, United States; 2 Health Department, UNICEF Headquarters New York, Manhattan, NY, United States; 3 Health Department, UNICEF Geneva, Geneva, Switzerland; 4 World Health Organization Regional Office for Africa, Brazzaville, Democratic Republic of Congo; 5 Health Department, UNICEF East Asia Pacific Regional Office, Bangkok, Thailand

**Keywords:** digital documentation of COVID-19 certificates, immunization agenda 2030, immunization information systems, routine immunization, sustainable development goals

## Abstract

While most existing digital immunization systems lack mechanisms to capture high fidelity real-time data to respond to current needs; many others are not designed to support interoperability and data sharing across the continuum of care for the health Sustainable Development Goals (SDGs). In this paper, we used the World Health Organization (WHO) Digital Documentation of COVID-19 Certificates (DDCC) as a proxy to demonstrate and operationalize how an efficient digital immunization system could strengthen service delivery and optimize outcomes for the SDGs. This paper appraises the technical, ethical and cultural considerations for establishing DDCC and how it can be operationalized among national health systems. It demonstrates how digital health investments can support routine immunization for the SDGs and highlights the critical role global health leadership plays in shaping reforms for national digital transformation agenda. The adoption and institutionalization of digital immunization systems offer opportunities to bridge multiple information solutions and strengthen immunization service delivery towards sustainable outcomes for the SDGs. Thus, it is recommended that Development partners and implementers jointly work with governments to shape the national digital health ecosystem that connects multiple healthcare journeys for the sustainable immunization agenda 2030.

## Introduction

While most existing digital immunization systems lack mechanisms to capture high fidelity real-time data to respond to current needs; many others are not designed to share data across the continuum of care for the health Sustainable Development Goals (SDGs). The World Health Organization (WHO) and UNICEF in collaboration with the Digital Health Centre of Excellence (DICE) in 2022 developed and deployed the Digital Documentation of COVID-19 Certificates (DDCC) to address contextual complex challenges regarding access during COVID-19 lockdowns, and the certificates needed to access places (1–3).

The DDCC is a mechanism by which a person's COVID-19-related health data can be digitally documented through an electronic certificate for continuity of care, or as proof of vaccination for purposes other than healthcare (4). DDCC, which was originally designed as a digital COVID-19 certificate, has been expanded over time to cover all immunization records and the entire international patient summary that captures health events beyond routine immunization (4). As a digital health platform, DDCC contributes to the improvement of health systems by fostering interoperability and integration with national health/social registries and solutions with system-wide impacts (2, 5).

This paper moves the conversation from technical implementation of DDCC to a strategic, systemic, and equity-focused agenda. Using DDCC as a proxy for global Immunization Information System (IIS) reform, this paper highlights equity, financing, and cross-sector integration as critical enablers for sustainable immunization outcomes, embedding immunization systems into national digital transformation agendas. This paper reframes DDCC as a strategic lever for systemic digital health transformation— emphasizing equity, financing, cultural trust, and integration across sectors to achieve sustainable immunization outcomes for the SDGs. Digitizing immunization services is a priority for country-led national health systems as it facilitates seamless interconnection of multiple data systems facilitating robust data flows with optimal outcomes (6).

Recent evidence shows that digital immunization systems strengthen routine immunization by facilitating decision support systems, vaccine surveillance, logistics and supply chain management etc. (6–8),. Tozzi et al. (2016) documented vaccine confidence monitoring and the delivery of information related to vaccines to the public. They emphasized the use of digital platforms to develop tailored, evidence-based, and innovative strategies, tools, and messaging to increase vaccine confidence and uptake. This lends credence to the Vaccine Confidence and the Global Listening Project by Heidi Larson and colleagues (2022) that demonstrated the use of innovative digital platforms to send culturally sensitive messages, absolving hesitancy while addressing misinformation and misconceptions related to vaccine uptake and adherence (9). This aligns with WHO/GAVI/UNICEF's vision that pursues related strategies through partnerships, collaboration and digitization (3, 7, 10).

In the East Asia and Pacific Region, UNICEF, WHO and other partners have, through novel approaches, strengthened the use of digitization for key programmes that target broader health systems’ strengthening (11, 12). Through rights-based approaches, such digital coordinating mechanisms among Partners and Regional Networks have strengthened vaccine data systems, scaled deployment of Immunization Information Systems (IIS) and advanced immunization service delivery with system-wide impacts (3, 13). Thereby ensuring a digitally-enabled health system that is highly technologically enabled, while recognizing the persisting digital divide.

Digital inequities in the use and access of digital solutions persist and go beyond access to information and communication resources (14, 15). Recent evidence shows that in 2013, only 47% of the population among emerging and developing countries had access to the internet, whereas an estimated 87% of the population had access to the internet among developed countries in 2015 (16). Thus, bridging the digital divide is critical for a connected and sustainable digital society (17). Wang and Ping in 2022 did an assessment of COVID-19 vaccination across 12 countries. Their study documented a lack of connectivity and systemic integration among varied siloed systems that led to redundant efforts and poor outcomes. These are multiple standalone solutions that with no enterprise integrations and lack capabilities to share real-time data (5, 18, 19).

According to WHO, the rising pace of immunization information systems comes with opportunities and threats. Most of these solutions are standalone solutions with no systemic integrations for impacts (5) and are deployed in response to either a specific need or to support specific programme (3, 20). Gleiss and Lewandowski (2022) documented an increased loss of efficiency and productivity following deployment of multiple standalone solutions in hospitals and recommended the adoption of an integrated hospital data management platform with capabilities to share real-time data. A recent WHO report also documented the impacts of deploying fragmented solutions in countries. Such solutions were found to undermine governments' efforts on delivering quality services as they lack mechanisms to share data for the continuum of care with loss of efficiency (5, 19, 21). Thus, WHO have recommended the adoption and use of integrated IIS for routine immunization.

Immunization Information Systems represents a confidential, population-based, computerized information system that records, stores, and provides access to consolidated individual immunization information that is comprehensive and community-wide across multiple healthcare providers. Integrated IIS can offer other capabilities, such as reminder/recall notifications, vaccine supply and stock management, and adverse event reporting (21). Different IIS range in complexity and scope, with varied functionalities (22–24). Multiple reports have documented the critical role of IIS in strengthening routine immunization and have advocated the adoption and use of IIS among global health systems. However, most countries' programmes due to system-related challenges have deployed standalone immunization registries with no integration/interoperability frameworks for the broader health programmes' integrations with loss of efficiency, productivity and impact (11, 25).

The pertinent question is whether it is feasible to develop an integrated IIS with capabilities of addressing related challenges and strengthening immunization programmes for the SDGs. Yes, this is possible as exemplified by the WHO DDCC. However, there is a need to support countries to adopt, deploy, scale and institutionalize IIS. There is also a need to strengthen existing digital immunization systems to further support the recording and reporting of vaccine supplies to ensure it reaches the target population. For this to happen, more work on system strengthening and integrations needs to be done, and mechanisms must be put in place to support countries to transition from paper-based documentation to agile digital systems to ensure that vaccines are tracked and distributed equitably up to the last mile. Such mechanisms will ensure that governments are committed and are willing to be guided to prioritize deploying integrated digital immunization solutions (26).

### Bridging the gap: the use of DDCC as a proxy for strengthening immunization information systems

This section highlights the technical, ethical and cultural considerations of DDCC concepts, including its operationalization in countries. Interoperability^1^ of digital immunization systems is critical and should follow basic Open Health Information Exchange standards. DDCC architecture contains standards-compliant specifications that ensure robust interoperability with other systems. Through standard-based frameworks, DDCC is able to integrate and share data with Vaccine Delivery System, Electronic Medical Record, and Shared Health Record etc., to ensure continuity of care (2, 27). Broader system interoperability with national enterprise infostructure across other sectors (commerce, education, transportation, agriculture etc.,) is possible through robust digital public infrastructure (2). Such open standards and technologies ensure that healthcare information is securely available to serve continuity of care including proof of vaccination and other purposes beyond health (28).

As documented by WHO, electronic certifications (DDCC) should be made accessible to both the vaccinated individuals and health workers in different formats for continuity of care, or as proof of vaccination for purposes other than health care (27) and should be able to have online/offline flexibilities. The DDCC implementation guidance highlights critical pre-deployment and deployment considerations including consideration on cost/benefit analysis, security/privacy concerns, ethical/cultural validations and trust frameworks (2). These corroborate with the study by Mithani et al. (2021) that demonstrated how DDCC anti-fraud mechanisms helped establish certification integrity while dissuading systemic inequities (29).

At the country level, the development and implementation of DDCC is done with consultations with the end-users. Wang and Ping (2022) maintain that DDCC development should be communicated in a transparent manner to promote public trust and acceptance in-line with DDCC operational guidance. DDCC systems work to benefit individuals and the public health including supporting national policies to limit access to and use of a DDCC by third parties (18, 25). Thus, an ideal DDCC ensures that countries/individuals are not locked into commitment with only one vendor, rather countries, are responsible for distributing DDCC and any associated representation of the data, such as a QR code, to DDCC holders (2, 28).

Government-to-Government agreements are critical for sharing and validating DDCC internationally through an authentication key– Public Key Infrastructure (PKI) that is used in signing DDCC, and ensures information is correctly shared among countries. Countries with no PKI in place are supported to finalize their national use-case (2, 30, 31). Jose et al. (2021) maintained that when a PKI is added to DDCC architecture, it can be linked to the certifying authority, creating an internationally recognizable QR code while ensuring continuity of care.

Countries deployed DDCC with different experiences. The Philippines DDCC also known as “VaxCertPH” was a typical use-case. Data management and governance framework facilitated the generation of robust data for programme improvements (32). The Estonia DDCC was integrated into the routine national Health Management Information System (HMIS) for efficiency and effectiveness with inherent data quality checks in place for system/program improvements. Quality assurance mechanisms were put in place and ensured seamless collation of high-fidelity data with optimal output (33). This is akin to the Kenyan DDCC use-case where the enterprise architecture supported real-time data systems for efficient data analysis and reporting (34). The Canadian DDCC system has mechanisms to merge multiple vaccine records into a single master record to minimize data duplications from multiple requests (35). These country use-cases provide a seamless globalization of the WHO DDCC and representative of an integrated IIS for strengthening routine immunization (2, 18, 27, 36).

Moving forward, the WHO secretariat under the Global Strategy for Digital Health has established the Global Digital Health Certification Network (GDHCN) as a digital public infrastructure to meet the confluence of requests for leadership and support, and to further understand the need to build upon the successes from COVID-19 certificates for greater health systems resilience (37). This builds upon the global strategy on Digital Health launched in 2020 (38); the DDCC guidance published in 2021 (2); the Federated public trust repository piloted in 2022 (30), and the Global Initiative on Digital Health established in 2024 (38). This direction is critical and will be built upon the legacy of DDCC and lessons learned from country-level deployment experiences (2, 37–41).

### Digitizing routine immunization programmes: opportunities and perspectives

UNICEF flagship of digital health advocates that everyone in every country gains access to vaccines through strengthened health systems equipped with cold chains. Along with its partners, UNICEF supplies vaccines to 45% of the world's children under-five in over 100 countries (42, 43). UNICEF works with governments and stakeholders to engage communities, procure and distribute these vaccines, whilst keeping supplies safe and effective and ensuring affordable access for even the hardest-to-reach families (41). However, some 20 million children globally still miss out on vaccines annually. The poorest and marginalized children continue to be the least likely to access routine immunization (44, 45). According to a recent WHO/UNICEF Estimate of National Immunization Coverage (WUENIC), the number of zero-dose children^2^ (1.8 million) still remains high in the East Asia and Pacific (EAP) region– approximately 88% higher than the annual goal of achieving the 2030 Immunization Agenda target of reducing the number of zero-dose children by half by 2030 in the EAP region (46).

To address related challenges and close the gap, UNICEF East Asia and Pacific Regional Office is supporting the deployment of integrated IIS in selected countries in the Region. The IIS enterprise planning and architecture has capacity to integrate with the WHO Reference Classifications for effective knowledge representation and interoperability. Particularly, the International Classification of Disease 11 (ICD-11) supports the recording of adverse events following immunization and linking immunization to morbidity surveillance. Similarly, the International Classification of Functioning, Disability and Health (ICF) support the capturing of functional outcomes in the post-vaccination stage; and the International Classification of Health Interventions (ICHI) supports the coding of immunization interventions and service delivery (47). This investment aims to strengthen immunization systems through digital technologies and will support governments to close the gap of the 1.8 million zero-dose children in the EAP Region (46).

While it is expected that these investments will have positive impacts and outcomes, having robust indicators in place for monitoring, cost-effectiveness/benefit analysis would be needed. This includes efficient oversight, ensuring that expenditures are consistent with plans and results are measurable and contribute to change. Improved clarity on immediate coverage is one benefit but understanding inequities will make the difference. This would help create a picture of which vulnerable groups are left behind and why (12, 26). Thus, it is advised that Development partners work with governments to strengthen PHC performance through greater availability and use of regular, reliable, and accurate information. This will foster seamless access to data systems, strengthen interoperability frameworks, and track progress and performance for the SDGs (7). Analysis and use of such data will drive improvements, and ensure that people receive the right vaccine, at the right time, by the right team, and in the right place. Moving forward, Development partners will continue to provide intensified and comprehensive technical support, including capacity-building and quality-assurance to countries, and will contribute to developing roadmaps for implementing and strengthening IIS. Existing health management information systems, structures and policies within the broader HSS and PHC spectrum will be strengthened, and better operationalized to move beyond aspiration to implementation (11, 21, 26, 45).

### Moving forward: harnessing global health policies and reforms for strengthening national digital transformation agenda

This section highlights the application of global policies and resolution in strengthening national health systems through digitization, innovation, and partnerships. The COVID-19 pandemic has led to an acceleration of a process well underway before COVID-19 outbreak. These are processes that many had been advocating for nearly a decade—moving away from a state of discordant proliferation of pilot projects, few of which were capable of scale-up—towards true digital health system transformation, built on solid architectural foundations and driving towards a better, more equitable person-centered health journey. The pandemic necessitated the need for us to scrutinize how we, as a public health community, can do better, but also to identify investments necessary for both health system strengthening and pandemic resilience. Recent evidence suggests that Digital Health is central to achieving our global health targets, with equity-driven, universal person-centered care as our north star (48).

The pertinent question is whether countries are willing to collaborate and converge existing digital health investment and activities under a single overarching framework, and what would the contours of this framework be. Led by country's health sector leadership, there is a need to shift the narrative, as well as investments—from individual, projectized digital health initiatives to an integrated, enterprise approach that prioritizes digital transformation and takes into consideration enterprise planning and architecture. Additionally, digital transformation for health must also take into consideration the government's broader strategy, which includes other line-ministries and investments in shared digital public infrastructure including identity management and payment platforms (49).

In 2020, the UN Member States ratified the Global Strategy on Digital Health which should serve as the overarching framework. The contours of this framework are articulated in various guidelines, including the Digital Implementation Investment Guide (DIIG) published by WHO in 2020 (37, 50). These already outlined contours focus on core digital health building blocks and the broader enabling environment, including policy, governance, coordination, standards, infrastructure, human resources and financing. However, despite this framework and guidelines being established, there has been uneven progress across countries in operationalizing them (50). These should be priorities to countries, and should include costed-blueprints and action plans, donor alignment, and systematic inclusion of resourcing within national health accounts and investment plans (51, 52).

Thus, Global Leadership, including the G20 should plan for a corpus fund to support systematic planning and implementation of Digital Health, especially among low- and middle-income countries (LMICs). A corpus fund should first be framed within these parameters, as these investments, when successful, are much broader than just health. It is critical to make the case for such a fund to non-health sector stakeholders—including Cabinets, Ministries of Finance, etc., Investments in digital public infrastructure and digital health should be seen as a matter of national security, and as a key contributor to not only health outcomes but national socio- economic development (26). Digital public infrastructure has demonstrated significant return on investment across many sectors, as can be seen from India (49) and Estonia (53) use-cases. However, additional investment cases and costed-blueprints backed by solid evidence and speaking to this larger narrative are still required.

This includes investments in democratization and promotion of Digital Public Goods (DPGs). Many of the existing DPGs have been driven by donors and organizations operating out of the global north, or by a single country. To be true digital health public goods, the use-cases need to come from a variety of countries spread across multiple regions, be tested across multiple geographies, and be financially and technically supported by a diverse set of stakeholders. G-20 countries can consider (1) creating sufficient incentives to encourage diversification, including investing in partners across multiple geographies in the global south that can support existing digital public goods; 2) supporting countries to establish interoperability laboratories and sandboxes to lower the barrier of entry for local entrepreneurs. The India's sandbox+planned open Machine Learning libraries are a great example; 3) establishing incentives and business modeling to support the open-sourcing of promising proprietary platforms; 4) addressing financing gaps for digital public good core platform support, which is often neglected; and 5) ensuring that Request-For-Proposals promote and prioritize DPGs, and that they are being evaluated against clear criteria including common functionality, including the WHO clinical and public health guidelines, interoperability standards, cyber security and data protection (37, 50).

To achieve these, there is a need for a global consensus among International Organizations, Philanthropies, Civil Societies Organizations (CSOs) etc., to collaborate to bridge the gaps in achieving at-scale implementation of digital health solutions (including tele-healthcare solutions) and Innovations. Digital transformation must be led by the government, but to be successful—it will require an aligned well-coordinated approach including international organizations, CSOs, academia, the private sector, philanthropists etc. No single agency can succeed on its own, and misaligned digital investments can disproportionately weaken and fragment the entire ecosystem as well as put citizens at risk (54). The G-20 can consider endorsing not only a common vision and set of principles but a clear operational roadmap, and ensuring that loans, grants and their respective government development agencies champion (or at least adhere) to these guidelines and best practices. Additionally, existing professional networks—such as AeHIN, HELINA and RECAINSA– should continue to be strengthened, and capacity building—via national training institutions and universities—be prioritized.

The G20 countries should promote digital health innovations and become an enabler for the implementation of emerging technologies such as Artificial Intelligence, Machine Learning, Blockchain, IoTs, Drones, and geo-spatial mapping aiding disease surveillance and programme implementation within the G20 member states and amongst LMICs. G-20 countries should ensure that any investments in frontier technologies and digital health innovations address actual bottlenecks and country constraints, while not further extending the digital divide, marginalizing communities, or creating new risks for citizens. Additionally, before these innovations are endorsed or deployed at scale, sufficient evidence should exist. Many countries do not yet have policies or regulations around personal health data, standards, cyber security, etc., which should be a priority to ensure that innovations are in compliance with emerging best practices. Supported by a digital sandbox environment, new innovations can be systematically tested and adopted. Finally, to ensure that these new technologies are designed for the LMICs and mitigate as many risks as possible (including bias), open-source geo-spatial data, machine learning data libraries, etc., should be created across as many country contexts as possible. This will also help democratize this space and create opportunities for local entrepreneurs.

## Conclusion

This paper contributes to ongoing global conversations on the need to harness innovative digital technologies to strengthen immunization service delivery and improve health outcomes for the SDGs. This paper used the WHO Digital Documentation of COVID-19 Certificates as a proxy to demonstrate and operationalize how an efficient digital immunization system could strengthen service delivery and optimize outcomes. It demonstrates how digital health investments can support routine immunization for the SDGs and highlights the critical role global health leadership plays in shaping reforms for national digital transformation agenda. We propose future studies to highlight country-specific digital immunization information/infrastructure gaps, along with discussion of the costs, benefits, and trade-offs of digital transformations in countries. Thus, we recommend Development partners and implementers to jointly work with governments to shape the national digital health ecosystem that connects multiple healthcare journeys for the sustainable immunization agenda 2030.

## Data Availability

The original contributions presented in the study are included in the article/Supplementary Material, further inquiries can be directed to the corresponding author.

## Footnotes

^1^According to WHO, interoperability is the ability of different information systems and applications to communicate, exchange data, and use the information that has been exchanged

^2^According to WHO, the term ‘zero-dose children’ is defined as those that lack access to or are never reached by routine immunization services

## References

[1] DICE. Digital Health Center of Excellence (2020). Available online at: https://www.digitalhealthcoe.org/ (Accessed March 11, 2025).

[2] WHO. Digital documentation of COVID-19 certificates: vaccination status: technical specifications and implementation guidance (2021). Available online at: https://www.who.int/publications/i/item/WHO-2019-nCoV-Digital_certificates-vaccination-2021.1 (Accessed February 21, 2025).

[3] UNICEF. UNICEF’s Digital Health & Information Systems Annual Report (2022). Available online at: https://www.digitalhealthcoe.org/knowledgebase/unicef%E2%80%99s-digital-health-%26-information-systems-annual-report-2022 (Accessed March 21, 2024).

[4] Hausam R D'Amore J Cangioli G Chronaki C Geßner C Macary F International Patient Summary Implementation Guide. Accessed from: Home - International Patient Summary Implementation Guide v2.0.0. (2025). (Accessed August 1, 2025).

[5] World Health Organization & International Telecommunication Union. Digital Health Platform Handbook: Building a Digital Information Infrastructure (Infostructure) for Health. Geneva: World Health Organization and International Telecommunication Union (2020). p. 1–125. Available online at: https://iris.who.int/server/api/core/bitstreams/4843e6f7-85a4-4a91-a8ba-64da6bf44b9d/content (Accessed August 20, 2025). License: CC BY-NC-SA 3.0 IGO.

[6] Menaechmi N Collum TH. Benefits and drawbacks of electronic health record systems. Risk Manag Healthc Policy. (2011) 4:47–55. 10.2147/RMHP.S12985 22312227 PMC3270933

[7] Tozzi AE Gesualdo F D’Ambrosio A Pandolfi E Agricola E Lopalco P. Can digital tools be used for improving immunization programs? Front. Public Health. (2016) 4:36. 10.3389/fpubh.2016.00036 PMC4782280 27014673

[8] Alscher A Schnellbächer B Wissing C. Adoption of digital vaccination services: it is the click flow, not the value—an empirical analysis of the vaccination management of the COVID-19 pandemic in Germany. Vaccines (Basel). (2023) 11(4):750. 10.3390/vaccines11040750 37112662 PMC10145467

[9] Larson H. Finding Exemplars of Restored Vaccine Confidence: A Pathway for COVID-19 Recovery (2020). Available online at: https://blogs.worldbank.org/en/health/finding-exemplars-restored-vaccine-confidence-pathway-covid-19-recovery (Accessed June 12, 2025).

[10] Garrett PM White JP Dennis S Lewandowsky S Yang CT Okan Y Papers please—predictive factors of national and international attitudes toward immunity and vaccination passports: online representative surveys. JMIR Public Health Surveill. (2022) 8(7):e32969. 10.2196/32969 35377317 PMC9290331

[11] UNICEF. UNICEF Annual Report 2020: Turning research into action for children and young people (2020). Available online at: https://reliefweb.int/report/world/unicef-annual-report-2020-turning-research-action-children-and-young-people (Accessed April 27, 2025).

[12] WHO. Primary health care measurement framework and indicators: monitoring health systems through a primary health care lens (2022). Available online at: https://www.who.int/news-room/events/detail/2022/02/28/default-calendar/launch-of-the-framework-and-indicators-for-monitoring-primary-health-care (Accessed March 25, 2025).

[13] UNICEF. UNICEF’s approach to digital health (2018). Available online at: https://www.unicef.org/innovation/reports/unicefs-approach-digital-health%E2%80%8B%E2%80%8B (Accessed January 16, 2025).

[14] Acilar A Sæbø Ø. Towards understanding the gender digital divide: a systematic literature review. Glob Knowl Memory Commun. (2023) 72(3):233–49. 10.1108/GKMC-09-2021-0147

[15] Markri A. The lancet digital health: bridging the digital divide in health care. Lancet. (2019) 1(5):204. 10.1016/S2589-7500(19)30111-6

[16] Poushtre J. Smartphone ownership and internet usage continues to climb in emerging economies, but advanced economies still have higher rates of technology use (2016). Available online at: https://www.diapoimansi.gr/PDF/pew_research%201.pdf (Accessed April 26, 2025).

[17] Vassilakopoulou P Hustad E. Bridging digital divides: a literature review and research agenda for information systems research. Inf Syst Front. (2023) 25:955–69. 10.1007/s10796-020-10096-3 33424421 PMC7786312

[18] Wang B Ping Y. A comparative analysis of COVID-19 vaccination certificates in 12 countries/regions around the world: rationalizing health policies for international travel and domestic social activities during the pandemic. Health Policy. (2022) 126(8):755–62. 10.1016/j.healthpol.2022.05.016 35680529 PMC9148623

[19] Ibeneme S Karamagi H Muneene D Goswami K Chisaka N Okeibunor J. Strengthening health systems using innovative digital health technologies in Africa. Front Digit Health. (2022) 4:854339. 10.3389/fdgth.2022.854339 35434700 PMC9008130

[20] Moore C Werner L BenDor AP Bailey M Khan N. Accelerating harmonization in digital health. J Health Population. (2021) 17:43–54. 10.12927/whp.2017.25306 29400273

[21] Gleiss A Lewandowski S. Removing barriers for digital health through organizing ambidexterity in hospitals. J Public Health. (2022) 30:21–35. 10.1007/s10389-021-01532-y

[22] ECDE. ECDC launches a handbook on the design and implementation of immunization information systems (2018). Available online at: https://www.ecdc.europa.eu/en/news-events/ecdc-launches-handbook-design-and- implementation-immunisation-information-systems (Accessed April 13, 2025).

[23] CDC. About Immunization Information Systems (2019). Available online at: https://www.cdc.gov/vaccines/programs/iis/about.html (Accessed April 20, 2025).

[24] European Union. EU Digital COVID certificate. The European Union (2021). Available online at: https://commission.europa.eu/strategy-and-policy/coronavirus-response/safe-covid-19-vaccines-europeans/eu-digital-covid-certificate_en (Accessed March 21, 2025).

[25] Zhu DT Serhan M Mithani SS Smith D Ang J Thomas M The barriers, facilitators and association of vaccine certificates on COVID-19 vaccine uptake: a scoping review. Global Health. (2023) 19(1):73. 10.1186/s12992-023-00969-y 37759306 PMC10537206

[26] United Nations. Sustainable Development Goals: Ensure Healthy Lives and Promote Well-Being for all at all Ages. (2019). Available online at: https://unstats.un.org/sdgs/report/2019/goal-03/ (Accessed March 17, 2025).

[27] Voo TC Smith MJ Mastroleo I Dawson A; WHO Ethics & COVID-19 Working Group. COVID-19 vaccination certificates and lifting public health and social measures: ethical considerations. East Mediterr Health J. 2022;28(6):454–8. 10.26719/emhj.22.023 35815877

[28] Jackson E.B Dreyling R. Pappel I. (2021). Challenges and implications of the WHO’s digital cross-border COVID-19 vaccine passport recognition pilot. Eighth International Conference on EDemocracy & EGovernment (ICEDEG), Quito, Ecuador, 2021, pp. 88-94, 10.1109/ICEDEG52154.2021.9530954

[29] Mithani SS Bota AB Zhu DT Wilson K. A scoping review of global vaccine certificate solutions for COVID-19. Hum Vaccin Immunother. (2022) 18(1):1–12. 10.1080/21645515.2021.1969849 34613869 PMC8920155

[30] Hernández-Ramos JL Karopoulos G Geneiatakis D Martin T Kambourakis G Fovino IN. Sharing pandemic vaccination certificates through blockchain: case study and performance evaluation. Wireless Communications and Mobile Computing. (2021) 2021:12. Article ID 2427896. 10.1155/2021/2427896

[31] Sharun K Tiwari R Dhama K Rabaan AA Alhumaid S. COVID-19 vaccination passport: prospects, scientific feasibility, and ethical concerns. Hum Vaccin Immunother. (2021) 17(11):4108–11. 10.1080/21645515.2021.1953350 34292128 PMC8828155

[32] CORESIA. A regional study on vaccination certificates - Philippines (2022). Accessed from: Available online at: https://vaxcert.info/?country=philippines-2&lang=en (Accessed March 30, 2025).

[33] CORESIA. A regional study on vaccination certificates - Estonia (2022). Available online at: https://vaxcert.info/?country=estonia-2&lang=en (Accessed March 20, 2024).

[34] CORESIA. A regional study on vaccination certificates - Kenya (2022). Available online at: https://vaxcert.info/?country=kenya-2&lang=en (Accessed March 11, 2025).

[35] CORESIA. A regional study on vaccination certificates - Canada (2022). Available online at: https://vaxcert.info/?country=canada-2&lang=en (Accessed March 17, 2025).

[36] de Miguel Beriain Í Rueda J. Digital COVID certificates as immunity passports: an analysis of their main ethical, legal, and social issues. J Bioeth Inq. (2022) 19(4):635–42. 10.1007/s11673-022-10209-4 36121608 PMC9484347

[37] WHO. Global Digital Health Certification Network (2023). Available online at: https://www.who.int/initiatives/global-digital-health-certification-network (Accessed June 21, 2025).

[38] WHO. Global strategy on digital health 2020-2025 (2021). Available online at: https://www.who.int/publications/i/item/9789240020924 (Accessed April 21, 2025).

[39] WHO. Global Index on Digital Health (2024). Available online at: https://www.who.int/initiatives/gidh (Accessed July 18, 2025).

[40] Jeyakumar JIH Walker J Roßnagel H. Decentralized global trust registry platform for trust discovery and verification of e-health credentials using TRAIN: cOVID-19 certificate use case. In: Rios R Posegga J, editors. Security and Trust Management. STM 2023. Lecture Notes in Computer Science, 14336. Cham: Springer (2023). p. 95–104. 10.1007/978-3-031-47198-8_6

[41] Godinho MA Liaw S Kanjo C Marin HF Martins H Quintana Y. Digital vaccine passports and digital health diplomacy: an online model WHO simulation. J Am Med Inform Assoc. (2023) 30(4):712–7. 10.1093/jamia/ocac126 35866622 PMC9384525

[42] Towett G Snead RS Grigoryan K Marczika J. Geographical and practical challenges in the implementation of digital health passports for cross-border COVID-19 pandemic management: a narrative review and framework for solutions. Global Health. (2023) 19:98. 10.1186/s12992-023-00998-7 38066568 PMC10709942

[43] UNICEF. Annual reports on UNICEF’s results for children 2023 (2023). Available online at: https://www.unicef.org/reports/country-regional-divisional-annual-reports Accessed April 25, 2025.

[44] UNICEF. UNICEF Annual Report 2022 (2022). Available online at: https://www.unicef.org/reports/unicef-annual-report-2022 (Accessed April 23, 2025).

[45] UNICEF. UNICEF East Asia and the Pacific Regional Office Annual Report 2023 (2023). Available online at: https://www.unicef.org/eap/media/13611/file/UNICEF%20Annual%20Report%202022% 20.pdf (Accessed April 26, 2025).

[46] WHO/UNICEF. Immunization Analysis and Insights (2025). Available online at: https://www.who.int/teams/immunization-vaccines-and-biologicals/immunization-analysis-and-insights/global-monitoring/immunization-coverage/who-unicef-estimates-of-national-immunization-coverage (Accessed August 09, 2025).

[47] World Health Organization. Classifications and terminologies (2024). Available online at: https://www.who.int/standards/classifications (Accessed December 30, 2025).

[48] Petracca F Ciani O Cucciniello M Tarricone R. Harnessing digital health technologies during and after the COVID-19 pandemic: context matters. J Med Internet Res. (2020) 22(12):e21815. 10.2196/21815 33351777 PMC7775375

[49] Kaur S Mir SA. Digital India: an analysis of its impact on economic, social, and environmental sectors. NeuroQuantol. (2022) 20(22):2551–61. 10.48047/NQ.2022.20.22.NQ10246

[50] World Health Organization. Digital implementation investment guide (DIIG): integrating digital interventions into health programmes. World Health Organization. (2020). p. 1–182. Available online at: https://www.who.int/publications/i/item/9789240010567 (Accessed April 23, 2024).

[51] Ibeneme S Revere L Okeibunor J Hwang L Muneene D Ukor N. Strengthening capacities among digital health leaders for the development and implementation of national digital health programs in Nigeria. BMC Proc. (2020) 14(Suppl 10):9. 10.1186/s12919-020-00193-1 32714444 PMC7376631

[52] Koch S Hersh W.R Bellazzi R Leong T.Y Yedaly M Al-Shorbaji N. Digital Health during COVID-19. (2021). Informatics. Dialogue with the World Health Organization. Available online at: https://www.thieme-connect.com/products/ejournals/pdf/10.1055/s-0041-1726480.pdf (Accessed April 23, 2025). 10.1055/s-0041-1726480 PMC8416211 33882596

[53] Białczyk A Leśniak G Nadolny F Mrowiec J Otałęga A. Exploring digital health horizons: a narrative review of E-health innovations in Poland, Spain, Romania and Estonia. Prospects in Pharmaceutical Sciences. (2024) 22(1):32–7. 10.56782/pps.178

[54] Olu O Muneene D Bataringaya JE Nahimana M Ba H Turgeon Y How can digital health technologies contribute to sustainable attainment of universal health coverage in Africa? A perspective. Front Public Health. (2019) 7:341. 10.3389/fpubh.2019.00341 31803706 PMC6873775

